# Crosslinked Waterborne Polyurethane Solid–Solid Phase Change Materials with Polyethylene Glycol for Thermoregulating Textiles

**DOI:** 10.3390/polym18141712

**Published:** 2026-07-12

**Authors:** Hongjie Cao, Yanli Sun, Shaofeng Lu, Bo Li, Songcong Lin, Qiancheng Yang, Chengcheng Tian, Fan Zhang

**Affiliations:** 1 School of Textile Science and Engineering, Xi’an Engineering University, Xi’an 710048, China; 250111013@stu.xpu.edu.cn (H.C.); sunyanli@xpu.edu.cn (Y.S.); 13990105588@126.com (Q.Y.); tiancheng246@163.com (C.T.); zhangfan@xpu.edu.cn (F.Z.); 2 Key Laboratory of Functional Textile Material and Product, Xi’an Polytechnic University, Ministry of Education, Xi’an 710048, China; 3 Key Laboratory of Functional Apparel Fabrics of Shaanxi Province, Xi’an 710048, China; 4 Fujian Techwork Textile Co., Ltd., Quanzhou 362700, China; 13599128855@126.com

**Keywords:** polyurethane, solid–solid phase change material, cross-network structure, thermoregulating textile

## Abstract

Solid–solid phase change materials integrate high heat storage, leak-proof performance, and dimensional stability, thereby positioning them as optimal candidates for thermoregulating textile applications. This study effectively synthesized PEG-based crosslinked waterborne polyurethane phase change materials (WPU-SSPCMs) with polyethylene glycol (PEG) as the soft segment, hexamethylene diisocyanate (HMDI) as the diisocyanate, and glycerol (GL) as the multifunctional chain extender. The effects of PEG molecular weight, diisocyanate type, chain-extension temperature, and monomer molar ratio on the phase transition behavior and thermal stability of WPU-SSPCMs were systematically investigated. The results indicate that at a PEG molecular weight of 2000, a chain-extension temperature of 70 °C, and a monomer molar ratio of n(PEG:HMDI:GL) = 1:2:0.67, the synthesized WPU-SSPCMs exhibited optimal comprehensive performance. The material demonstrated a melting temperature of 33.38 °C with a high enthalpy of 80.31 J/g. It displayed characteristic solid–solid phase transition behavior without evidence of liquid leakage during heating. Following 100 thermal cycles, the enthalpy variation remained below 1 J/g, indicating exceptional thermal and cycling stability. When applied to cotton fabric, the resultant thermoregulating textile displayed a buffering plateau during both heating and cooling processes, demonstrating a pronounced temperature-regulating effect relative to untreated fabric. The developed WPU-SSPCM holds considerable promise for applications in intelligent temperature regulation.

## 1. Introduction

With the rapid development of global industrialization and technology, the shortage of natural resources and the environmental pollution caused by industrial production have become critical constraints on global economic growth and technological development [1,2]. Therefore, the development of novel energy-saving materials has become urgent and essential [3]. Among various energy storage materials, phase change materials (PCMs) have attracted considerable research interest, owing to their environmentally friendly characteristics, high energy storage density, and excellent reversibility, making them promising candidates for sustainable thermal energy storage applications. PCMs are functional materials that can undergo phase transition within a specific temperature range, and can store or release thermal energy as the temperature changes [4].

Currently, the PCMs commonly employed for thermal energy storage are mainly divided into solid–liquid phase change and solid–solid phase change [5]. Solid–liquid phase change materials are widely used due to their high thermal storage density. However, these materials require complex encapsulation processes to avoid leakage during application, which not only enhances the production costs but also increases thermal resistance and affects the heat transfer efficiency of phase change materials [6]. In contrast, solid–solid phase change materials (SSPCMs) do not experience significant volume expansion or liquid leakage during application, making them more valuable for production and development.

Among these, polyurethane solid–solid phase change materials (PU-SSPCMs) have garnered considerable attention due to their high latent heat storage capacity, superior structural designability, and excellent thermal stability [7,8]. A typical PU-SSPCM comprises flexible soft segments (e.g., polyethylene glycol) and rigid hard segments (e.g., diisocyanate and chain extender), wherein phase transition temperature and enthalpy values can be precisely modulated by adjusting the ratio or molecular structure of the soft and hard segments [9,10]. Furthermore, PU-SSPCMs exhibit solid–solid phase change characteristics, which effectively avoid the leakage issues seen in traditional solid–liquid phase change materials. Wei [11] synthesized a novel solid–solid phase change heat storage material through a two-step condensation reaction of PEG10000, PE, and MDI. The material’s phase change enthalpy is 152.97 kJ/kg, and the phase-change temperature is 58.68 °C. Li Liao [12] prepared a prepolymer by reacting PEG6000 with liquefied MDI and then synthesized a hyperbranched polyurethane solid–solid phase change material (HBPUPCM) using hyperbranched polyester (H20) as a chain extender. The results show that when the PEG content is between 70% and 90%, an HBPUPCM can be prepared with a phase-change temperature between 50.3 °C and 55.7 °C and a thermal enthalpy ranging from 78.4 J/g to 115.7 J/g. Nevertheless, most reported PU-SSPCMs are synthesized using organic solvent-mediated processes, which raise environmental and safety concerns during both material preparation and subsequent application. Moreover, their phase transition temperatures commonly surpass the 40–50 °C threshold, thereby exceeding the thermoneutral range for human physiological comfort (approximately 25–35 °C) and precluding their direct integration into thermoregulating fabrics [13,14]. Consequently, the development of eco-friendly synthetic routes, the reduction in phase transition temperatures to approximate ambient conditions, and the optimization of immobilization techniques onto flexible substrates are imperative [15].

In contrast, waterborne polyurethane (WPU), which utilizes water as the dispersion medium, exhibits salient advantages such as non-toxicity, absence of volatile organic solvents, and environmental friendliness [16,17]. Furthermore, its high compatibility with conventional textile finishing processes (e.g., dipping and padding) renders it more suitable for the practical production of smart thermoregulating textiles [18,19]. In thermoregulating textile applications, SSPCMs should not only possess high latent heat but also exhibit a phase-change temperature close to the human comfort temperature range (approximately 28–37 °C). Therefore, considerable efforts have recently been devoted to tailoring the phase-change temperature of polyurethane-based SSPCMs through molecular structure design. Wang [20] reported that the transition temperature of polyurethane SSPCMs could be effectively regulated by adjusting the molecular weight and blending ratio of polyethylene glycol (PEG), providing a flexible strategy for designing materials with tunable phase-change temperatures. Similarly, Zhou [4] developed hyperbranched waterborne polyurethane SSPCMs by optimizing the PEG content and hyperbranched network structure, achieving a phase-change temperature suitable for thermoregulating textile applications while maintaining excellent thermal cycling stability and temperature-regulation performance. Although these studies have successfully achieved suitable phase-change temperatures, textile materials are also expected to possess excellent flexibility, softness, and wearing comfort. However, increasing crystallinity or crosslinking density to improve thermal energy storage often leads to increased material stiffness, making it challenging to simultaneously balance phase-change temperature, latent heat, and flexibility. Therefore, developing polyurethane-based SSPCMs with both suitable phase-change temperatures and excellent flexibility remains an important challenge for thermoregulating textiles.

In this work, flexible waterborne polyurethane-based SSPCMs (WPU-SSPCMs) with suitable phase-change temperatures were developed by employing PEG as the soft segment, HMDI as the isocyanate, and glycerol (GL) as a multifunctional chain extender to construct a crosslinked polyurethane network. By regulating the molecular weight of PEG and the monomer composition, the phase-change temperature, latent heat, and flexibility of the WPU-SSPCMs were optimized simultaneously to meet the requirements of thermoregulating textile applications.

## 2. Experiment

### 2.1. Materials

Isophorone diisocyanate (IPDI, industrial grade) was provided by Guangdong Haoyi Chemical Technology Co., Ltd. (Guangdong, China); 4,4′-dicyclohexylmethane diisocyanate (HMDI, guaranteed reagent) was supplied by Xuzhou Yihuiyang New Materials Co., Ltd. (Xuzhou, China). Polyethylene glycol (PEG, analytical reagent) was purchased from the Tianjin Damao Chemical Reagent Factory (Tianjin, China). Glycerol (GL, analytical reagent) was supplied by Tianjin Fuyu Fine Chemical Co., Ltd. (Tianjin, China), and trimethylolpropane (TMP, analytical reagent) was obtained from the Tianjin Fuchen Chemical Reagent Factory (Tianjin, China). N,N-Dimethylformamide (DMF, analytical reagent) was supplied by Tianjin Fuyu Fine Chemical Co., Ltd. (Tianjin, China).

### 2.2. Synthesis of WPU-SSPCMs

**Figure 1:**
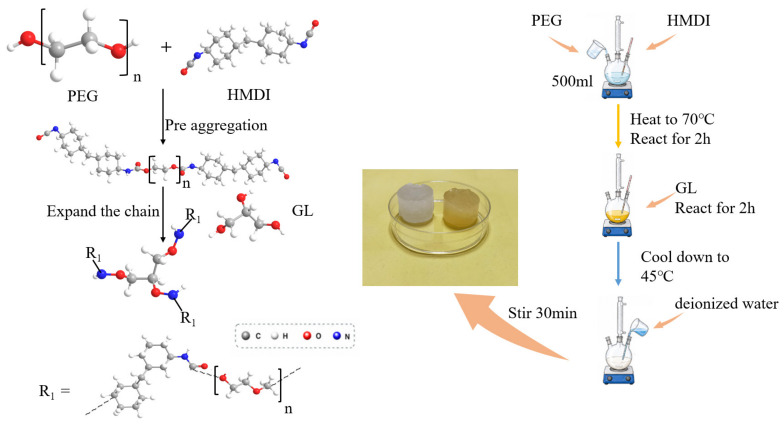
A schematic diagram of the synthesis process for the PEG-based crosslinked WPU-SSPCMs.

WPU-SSPCMs were synthesized using PEG, HMDI, and GL. Initially, the materials required pretreatment. HMDI, DMF and GL were dehydrated with 5A molecular sieves for more than 5 days, while PEG was vacuum-filtered at 120 °C for 2 h to remove water and other small molecules. Subsequently, 30 g of pretreated PEG was added into a 500 mL three-necked flask, followed by the addition of an appropriate amount of DMF with thorough mixing. The reaction system was heated to 50 °C, at which point a predetermined amount of HMDI was introduced. The temperature was then gradually raised to 70 °C, and the mixture was maintained under these conditions for 2 h to obtain the polyurethane prepolymer. Thereafter, GL was added to the solution to extend the chain for an additional 2 h. Throughout the reaction, sufficient DMF was added to alleviate the increase in viscosity resulting from system thickening. Finally, once the system was cooled to 45 °C, deionized water was added, and stirring continued for 30 min to afford WPU-SSPCMs. The synthesis process is illustrated in Figure 1.

### 2.3. Fabric Finishing

We prepared WPU-SSPCM solutions with different mass fractions and immersed cotton fabrics in these solutions for 30 min. Subsequently, the cotton fabric was subjected to continuous impregnation and rolling treatment twice, and then placed in a 110 °C oven for drying.

### 2.4. Characterization

The chemical structure of the prepared samples was characterized using an Spectrum TWO FTIR spectrometer (PerkinElmer, Waltham, MA, USA). over a wavenumber range of 4000–500 cm^−1^. The crystalline morphology during the phase transition process of PEG and WPU-SSPCMs was observed using DM2700P polarized light microscopy (POM) (Leica Microsystems, Wetzlar, Germany). Test samples were placed on an alcohol-cleaned glass slide, which was then positioned on the DM2700P for hot-stage POM analysis. The temperature was elevated to 70 °C and maintained for 20 min before being reduced to room temperature. The thermal properties of the samples were measured using a TAQ100 differential scanning calorimeter (DSC) (Mettler Toledo, Columbus, OH, USA). The test temperature range was −20 to 80 °C, with a heating and cooling rate of 5 °C/min under N_2_ protection. The thermal stability of the samples was evaluated via a thermogravimetric analyzer (TG) over a temperature range of 20–600 °C at a heating rate of 10 °C·min^−1^ under a N_2_ atmosphere. The crystallization behavior of WPU-SSPCMs was characterized via a Rigaku SmartLab SE X-ray (Rigaku Corporation, Japan, Tokyo) diffractometer employing a Cu Kα source over an angular range of 10–50° (2θ) at a scan rate of 5°/min. The fabricated materials were molded into standardized specimens and subjected to thermal treatment in an oven at a temperature exceeding their phase transition temperature. Following an isothermal hold of 20 min, the specimens were extracted. Their macroscopic morphological alterations were observed and documented through photographic imaging.

The determination of isocyanate (-NCO) content serves as a key indicator of reaction progression during the synthesis of WPU-SSPCMs. In the present work, this parameter was quantified via the dibutylamine titration method. A small amount of the reaction mixture was periodically sampled and transferred to a conical flask. A measured volume of dibutylamine–toluene solution was added using a pipette (DLAB Scientific Co., Ltd., Beijing, China), followed by rinsing with anhydrous toluene. The mixture was heated slowly under magnetic stirring and maintained for 30 min. Subsequently, 3–4 drops of bromocresol green indicator were added, and the solution was titrated with hydrochloric acid. Titration was terminated upon a sharp color change from blue to yellow, indicating the endpoint. The consumed volume of HCl was recorded as ***V*_1_**. A blank test was conducted under the same conditions. The -NCO content was calculated using Equation (1).

(1) $$-NCO\%=\frac{0.042\left(V_{2}-V_{1}\right)c}{m}\times 100\%$$

where ***V*_2_** denotes the volume of HCl consumed in the blank test (mL), ***V*_1_** denotes the volume of HCl consumed by the sample (mL), c represents the concentration of the standard HCl solution (mol/L), and m signifies the mass of the sample (g).

The surface morphologies of the untreated and thermoregulating fabrics were observed using a scanning electron microscope (SEM) (KYKY Technology Co., Ltd., Beijing, China). Prior to observation, all samples were sputter-coated with gold. To assess the thermoregulatory performance of the fabric, it was subjected to a thermal cycle comprising heating from −10 °C to 50 °C, an isothermal hold at 50 °C for 10 min, and then cooling to −10 °C. The fabric temperature was continuously monitored and recorded in real time using a digital thermometer (Hengshui Chuangji Instrumentation Co., Ltd., Hengshui, China). Meanwhile, the washing durability of the thermoregulating fabric was assessed in accordance with the relevant literature [20,21]. The enthalpy values of the fabric after successive washing cycles were measured via DSC, and the enthalpy retention rate (***ERR***) was calculated according to Equation (2):

(2) $$ERR(\%)=\frac{H_{1}}{H_{2}}\times 100$$

where ***H*_1_** (J/g) is the phase change enthalpy of the thermoregulating fabric after laundering, and ***H*_2_** (J/g) is that of the thermoregulating fabric before laundering.

## 3. Results and Discussion

### 3.1. Synthesis Mechanism

As shown in Figure 1, during polymerization, the hydroxyl groups of PEG reacted with the isocyanate groups of HMDI to generate a prepolymer, which in turn promoted the formation of a three-dimensional polyurethane network. According to previous studies, the incorporation of multifunctional polyols into polyurethane systems can induce chemical crosslinking through reactions between excess hydroxyl groups and isocyanate groups, thereby forming a stable three-dimensional network structure and effectively restricting molecular chain mobility. For example, Zhang et al. [22] successfully synthesized crosslinked polyurethane SSPCMs using glycerol as a trifunctional chain extender and attributed the formation of the three-dimensional network to its multifunctional hydroxyl architecture. Similarly, Lee and Kim [23] prepared crosslinked polyurethane SSPCMs using hyperbranched polyols as crosslinkers and demonstrated that the resulting network effectively constrained polymer chain movement and prevented leakage during phase transition. Accordingly, GL was selected as the chain extender to conduct the chain-extension reaction and further establish a crosslinked network structure.

### 3.2. Effect of PEG Molecular Weight on WPU–SSPCMs Performance

**Figure 2:**
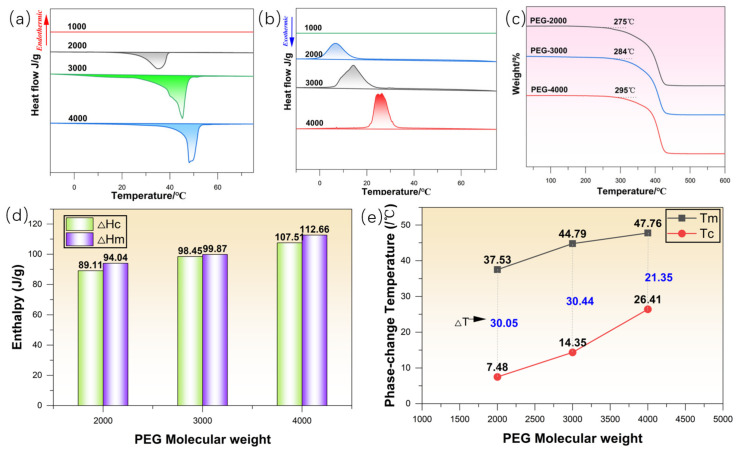
(**a**,**b**) DSC thermograms, (**c**) TGA curves, (**d**) phase change enthalpies and (**e**) phase-change temperatures of PEG-1000, PEG-2000, PEG-3000 and PEG-4000.

WPU–SSPCMs were synthesized using PEG with molecular weights ranging from 1000 to 4000, and the corresponding relationship between molecular weight and phase transition temperature, as well as phase transition enthalpy, was analyzed using DSC testing. The experimental results are presented in Figure 2a,b,d,e. When the PEG molecular weight was 1000, no obvious endothermic or exothermic peaks appeared in the DSC curve of WPU–SSPCMs, indicating that the material was in a state of incomplete crystallization. As the molecular weight of PEG gradually increased, the phase transition temperature and enthalpy of the synthesized WPU–SSPCMs also showed an increasing trend (Figure 2d). When the PEG molecular weight is 1000, its comparatively short polymer chains lack the capacity to establish stable crystalline domains within the crosslinked matrix. Furthermore, the substantial steric hindrance introduced by the hard segment inhibits crystallite propagation, thereby precluding any measurable phase change behavior. Conversely, PEG 2000–PEG 4000 possess sufficient chain length to form stable crystalline regions, substantially enhancing crystallinity and phase change performance. This trend is corroborated by prior studies, such as Yin et al. [24], who attributed the rise in phase transition temperature to the formation of more robust and complete PEG crystalline domains as the molecular weight increases. Extending the PEG chain length improves crystal integrity and intermolecular interactions, thereby increasing the thermal energy required to disrupt the ordered lattice during melting. Accordingly, the phase transition temperature ascends with PEG molecular weight. Additionally, higher PEG molecular weight augments the crystallization propensity of the soft segment. Fan et al. [25] observed that high-molecular-weight PEG displays stronger crystallization because longer chains exhibit greater conformational regularity and more readily assemble into ordered lattices. Hence, increasing PEG molecular weight elevates both crystallinity and latent heat storage capacity. Consequently, the crystallization area of WPU–SSPCMs increases, and there are changes in the enthalpy value and the phase transition temperature [26]. As shown in Figure 2e, when the molecular weights of PEG were 3000 and 4000, the melting temperatures of WPU–SSPCMs could reach 44.79 °C and 47.76 °C, respectively, which were not suitable for the development of temperature-regulating textiles [27]. Therefore, in order to meet the application requirements in the textile field, the PEG molecular weight of 2000 should be selected, and the phase transition temperature of WPU–SSPCMs synthesized under this condition is relatively low.

**Figure 3:**
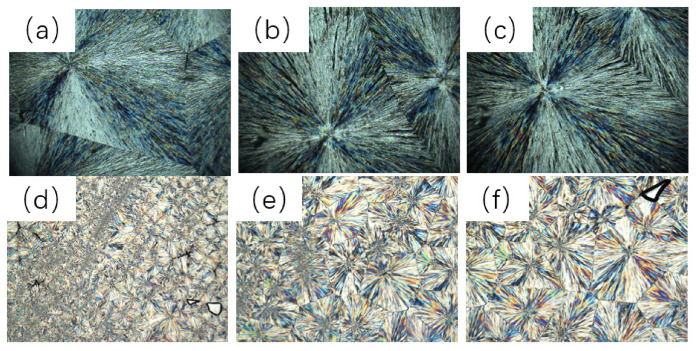
POM images of PEGs and corresponding WPU–SSPCMs based on different PEG molecular weights: (**a**) PEG 2000, (**b**) PEG 3000, (**c**) PEG 4000, (**d**) WPU–SSPCM_S_ 2000, (**e**) WPU–SSPCM_S_ 3000, and (**f**) WPU–SSPCM_S_ 4000.

Under normal temperature conditions (25 °C), the crystalline morphology of PEG with molecular weights ranging from 2000 to 4000 and their synthesized WPU–SSPCMs are observed using POM, as shown in Figure 3. It can be observed that in the POM test results of different molecular weight PEG and WPU–SSPCMs, distinct black cross extinction patterns are clearly observed. This indicates that all the samples exhibit a spherical crystalline morphology and that the crystalline phase of PEG remained unaltered during the synthesis of the WPU–SSPCMs. The results further reveal that, relative to pristine PEG of equivalent molecular weight, the crystallite dimensions of WPU–SSPCMs incorporating PEG as the soft segment are substantially reduced. This reduction is ascribed to the incorporation of rigid segments, which constrain the free mobility of the PEG chains. Consequently, the crystallization domains formed during the phase transition become more confined, leading to diminished crystallizability. As a result, crystal growth is restricted to a more limited spatial regime, yielding markedly smaller crystallites in WPU–SSPCMs compared to pure PEG [22].

Simultaneously, thermogravimetric analysis was performed on WPU-SSPCMs prepared with different PEG molecular weights, as illustrated in Figure 2c. It can be observed from Figure 2c that the thermal decomposition behavior of WPU-SSPCMs with varying PEG molecular weights is essentially consistent, with no significant weight loss occurring below 250 °C [28]. As the PEG molecular weight increased, the initial decomposition temperatures of the three WPU-SSPCMs also slightly increased, reaching 275 °C, 284 °C, and 295 °C, respectively. Therefore, all three WPU-SSPCMs demonstrated adequate thermal stability over their respective phase transition temperature ranges. Taking both thermal energy storage performance and thermal stability into account, PEG2000 was determined to be the most suitable soft segment for fabricating WPU-SSPCMs.

### 3.3. The Effect of Isocyanate Type on WPU-SSPCMs Performance

The hard segment structure is a critical factor influencing the performance of WPU-SSPCMs, and the molecular structure of distinct isocyanates fundamentally governs the configuration of the hard segment. Two aliphatic isocyanates with moderate reactivity, namely IPDI and HMDI, were selected to react with PEG2000 for the preparation of WPU-SSPCMs to investigate the effect of isocyanate structure on the properties of PCMs.

**Figure 4:**
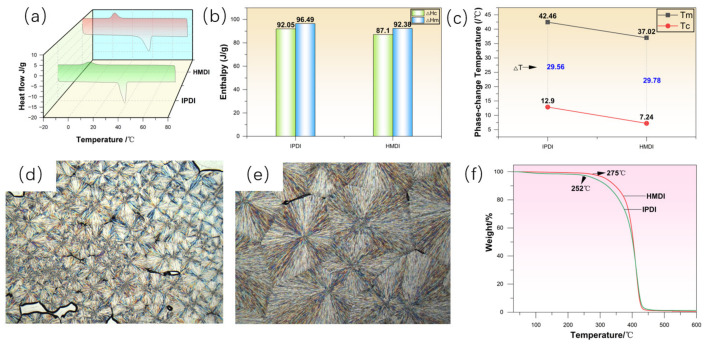
(**a**) DSC thermograms, (**b**) phase –change enthalpies, and (**c**) phase–change tempera-tures of IPDI and HMDI. (**d**) HMDI–based WPU–SSPCM_S_, (**e**) IPDI–based WPU–SSPCM_S_, and (**f**) comparison of their thermal stability.

Figure 4a–c present the DSC results of the HMDI- and IPDI-based WPU-SSPCMs. Although the enthalpy value of the HMDI-derived sample is marginally lower than that of its IPDI-based counterpart, its phase transition temperature is markedly reduced, indicating that HMDI imposes stronger constraints of the hard segments on the soft segments. From a molecular structure perspective, HMDI contains two six-membered carbon rings and exhibits high structural symmetry, whereas IPDI possesses only a single six-membered carbon ring. Consequently, HMDI generates greater steric hindrance during polymerization, resulting in reduced degrees of molecular chain freedom and enhanced confinement of the soft segments by the hard segments. This suppresses the crystallization performance of the soft segments, thereby yielding a lower phase transition temperature [28].

The crystalline morphology of WPU-SSPCMs synthesized from two distinct isocyanates was examined. As depicted in Figure 4d,e, POM images of both HMDI-based and IPDI-based WPU-SSPCMs display distinct black cross extinction patterns, confirming their crystalline state at ambient temperature. Nevertheless, the crystal dimensions of HMDI-based WPU-SSPCMs are markedly smaller than those of the IPDI-based variant. This disparity arises because the bicyclic architecture of HMDI diminishes the flexibility and conformational freedom of the molecular chains, thereby intensifying the constraint imposed by the hard segments on the soft segments, which restricts the crystalline growth of the latter and consequently yields finer crystal sizes [29].

As shown in Figure 4f, the thermogravimetric curves of HMDI- and IPDI-based WPU-SSPCMs exhibit no significant mass loss below 250 °C, indicating favorable thermal stability within their respective phase transition temperature ranges. The onset decomposition temperatures of the two samples are 275 °C (HMDI-based) and 252 °C (IPDI-based), with the HMDI-based sample demonstrating a notably higher value. This is attributed to the symmetric bicyclic structure of HMDI, which enhances molecular chain rigidity and elevates the required thermal decomposition temperature [7].

### 3.4. Effect of Chain-Extension Temperature on WPU-SSPCMs Performance

The chain-extension temperature constitutes a critical process parameter governing the subsequent reaction of the prepolymer and the crosslinking architecture of the final product. To elucidate its influence, three temperature conditions of 60 °C, 70 °C, and 80 °C were investigated. Zhou et al. [30] reported that the degree of polyurethane curing can be assessed by tracking the disappearance of the characteristic –NCO band during the curing process. The absence of this peak is generally regarded as evidence of substantial isocyanate consumption and successful polyurethane formation. Furthermore, Kim et al. [23] employed Fourier transform infrared spectroscopy (FTIR) to distinguish between fully cured and partially cured polyurethane coatings, demonstrating that the disappearance of the -NCO band is a reliable indicator of successful polyurethane network formation. Thus, by monitoring the –NCO content at various reaction intervals in conjunction with FTIR analysis, the study systematically investigated the effect of chain-extension temperature on the reaction extent of WPU-SSPCMs.

**Figure 5:**
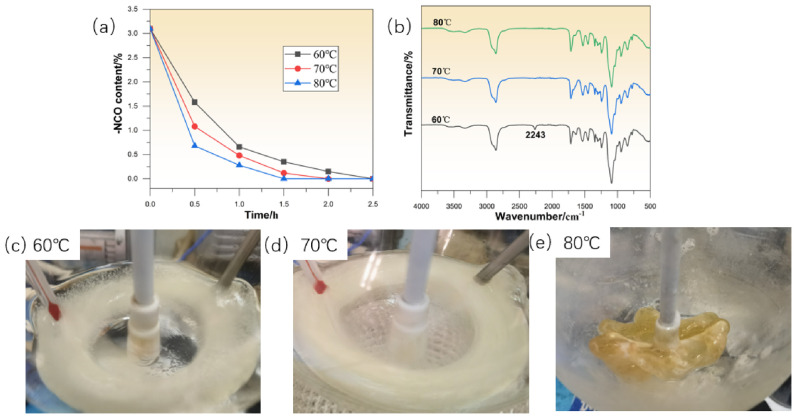
Effect of chain-extension temperature on the reaction behavior and chemical structure of WPU–SSPCMs: (**a**) reaction systems at different temperatures (60, 70, and 80 °C); (**b**) FTIR spectra of WPU–SSPCMs; and (**c**–**e**) degrees of reaction at different chain-extension temperatures.

The corresponding results are presented in Figure 5. The results illustrate the variation in –NCO content as a function of reaction time at different chain-extension temperatures. As shown, the –NCO content gradually decreased with prolonged reaction time across all tested temperatures, indicative of ongoing chain extension wherein –NCO groups and the hydroxyl groups of the chain extender are continuously consumed. Specifically, the times required for complete reaction at 70 °C and 80 °C are 2 h and 1.5 h, respectively, demonstrating that elevated temperature accelerates the chain-extension reaction rate [31]. Further analysis via FTIR, as depicted in Figure 5b, reveals that at the chain-extension temperature of 60 °C, the prepared WPU-SSPCM still exhibits a characteristic absorption peak of isocyanate (–NCO) at 2243 cm^−1^, indicating incomplete reaction of the –NCO groups. In contrast, when the chain-extension temperature is increased to 70 °C and 80 °C, this absorption peak vanishes, suggesting that the –NCO groups have undergone a complete reaction [32]. The disappearance of the characteristic –NCO absorption band at 2243 cm^−1^, together with the appearance of characteristic polyurethane bands, indicates that the isocyanate groups extensively reacted with hydroxyl groups to form polyurethane linkages. Evaluation of the reaction system at varying chain-extension temperatures (Figure 5c–e) revealed that at 60 °C, the process progressed steadily but was characterized by extended reaction duration and low efficiency. Upon increasing the temperature to 80 °C, a substantial rise in system viscosity was observed, accompanied by intensified reaction kinetics that rendered process control challenging and increased the propensity for uncontrolled polymerization. Based on a comprehensive assessment of conversion, system stability, and production efficiency, the optimal chain-extension conditions were established at 70 °C with a reaction time of 2 h.

### 3.5. Effect of Monomer Molar Ratio on the Properties of WPU–SSPCMs

The monomer molar ratio (PEG:HMDI:GL) is also the primary factor governing the performance of WPU–SSPCMs. Variations in this ratio alter the relative contents of soft and hard segments, thereby directly affecting the thermal storage capacity, phase transition behaviors, and crosslinking structure of the material. Accordingly, with a fixed amount of PEG, three molar ratios of n(PEG:HMDI:GL) were set at 1:1.5:0.33, 1:1.8:0.53, and 1:2:0.67 to systematically investigate the influence of the monomer ratio on the performance of WPU–SSPCMs.

**Figure 6:**
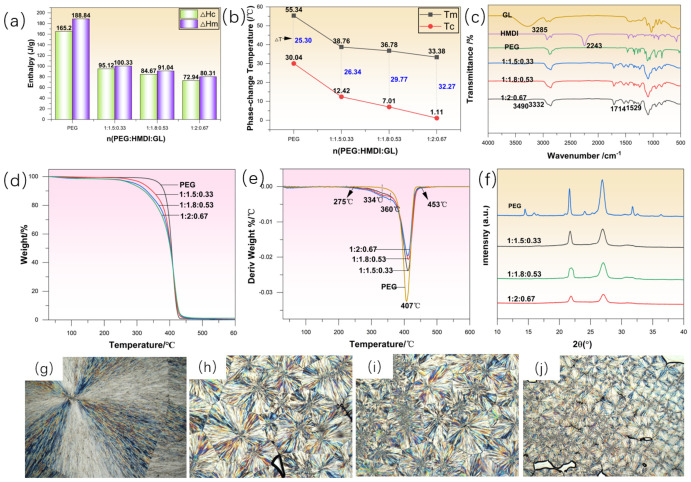
Effect of monomer molar ratio (n(PEG):n(HMDI):n(GL) = 1:1.5:0.33, 1:1.8:0.53, 1:2:0.67) on enthalpy, phase transition temperature, chemical structure, crystallinity, and thermal stability of WPU–SSPCMs: (**a**) phase change enthalpies, (**b**) phase-change temperatures, (**c**) FTIR spectra, (**d**) TG curves, (**e**) DTG curves, (**f**) XRD patterns, and POM images of (**g**) PEG, (**h**) WPU–SSPCMs–1.5, (**i**) WPU–SSPCMs–1.8, and (**j**) WPU–SSPCMs–2.0.

DSC results (Figure 6a,b) demonstrate that increasing the monomer molar ratio leads to a gradual decrease in the heat enthalpy of WPU-SSPCMs. This decline is attributable to the fact that the latent heat originates from the soft segment (PEG), whose relative content correspondingly decreases as the molar ratio rises. Additionally, both the Tm and Tc exhibit a downward trend, which is ascribed to an enhanced confinement effect exerted by the hard segments on the soft segments [33]. Notably, the Tc decreases more markedly with increasing molar ratio, resulting in a significant widening of the difference between the melting and crystallization temperatures (ΔT). This phenomenon arises because, confinement by the hard segments delays the growth time of spherulites, thereby depressing the crystallization temperature during the cooling process.

FTIR analysis (Figure 6c) demonstrated that the characteristic absorption band of HMDI at 2243 cm^−1^ and the hydroxyl band of GL at 3285 cm^−1^ were entirely absent in the products across all monomer molar ratios, suggesting that the isocyanate group of HMDI and the terminal hydroxyl groups of PEG and GL have completely reacted. Concurrently, the amide I band (C=O stretching vibration) at 1714 cm^−1^ and the amide II band (C-N stretching coupled with N-H deformation) at 1529 cm^−1^ appeared in the products. These two characteristic peaks are typical indicators of the polyurethane structure (-NHCOO-), proving that the obtained product is a polyurethane [33]. The characteristic peaks of the products prepared at varying monomer molar ratios were essentially identical, with no shifts observed in response to changes in the molar ratio. The absorption band within the 3332–3490 cm^−1^ range was attributed to the N-H stretching vibration. As the monomer molar ratio increased, both the hard segment content and the number of N-H groups increased, leading to a gradual rise in the integrated area of this absorption band. Simultaneously, the increase in hard segment content also resulted in a greater abundance of -NHCOO- linkages, evidenced by progressively larger peak areas for the amide I band at 1714 cm^−1^ and the amide II band at 1529 cm^−1^.

Figure 6d,e depict the TG and DTG curves of WPU-SSPCMs with varying monomer molar ratios. The TG curves demonstrate negligible mass loss below 275 °C for all samples, thereby confirming excellent thermal stability within the phase-change temperature range. Accordingly, the DTG curves reveal three decomposition stages. The first stage (275–334 °C) is associated with the scission of urethane linkages within the hard segments. As the monomer molar ratio increases, the hard-segment content and the number of urethane bonds increase, resulting in a progressively higher mass loss rate during this stage. The second stage (334–360 °C) involves the concurrent degradation of both hard segments and soft PEG segments. The third stage (360–453 °C) is ascribed to the decomposition of the PEG main chain within the soft segments, with the maximum degradation rate occurring at approximately 407 °C [34].

To further elucidate the crystallization behavior of WPU-SSPCMs, the crystal morphology and crystalline structure of PEG2000 and WPU-SSPCMs were characterized at ambient temperature via POM and XRD, with the corresponding micrographs presented in Figure 6h–j. The POM images reveal that elevating the monomer molar ratio induces a pronounced reduction in the crystallite dimensions of WPU-SSPCMs. On one hand, the augmented hard segment content exerts stronger confinement on the soft segments, restricting the mobility of the soft-segment molecular chains and thereby impeding crystallization growth. On the other hand, the heightened crosslink density compels crystallites to undergo mutual compression and collision during growth within a constrained spatial environment, leading to incomplete crystallization and a further decrease in crystallite size [29]. As depicted in Figure 6f, both pure PEG and the WPU-SSPCM exhibit prominent characteristic diffraction peaks at 21.61° and 26.87° at ambient temperature, confirming that both materials exist in a crystalline state with identical crystal morphology. Nonetheless, the intensity of these diffraction peaks is markedly diminished for the WPU-SSPCM relative to pure PEG, indicating that the crystallization of the PEG is hindered by the presence of hard segments. This observation further corroborates the restrictive effect of hard segments on soft segment crystallization. The results are consistent with previous studies on polyurethane-based solid–solid phase change materials. Wang et al. [16] reported that the spherulites of crosslinked polyurethane phase change materials are smaller than those of their linear counterparts, indicating that the crosslinked network imposes constraints on crystal growth.

### 3.6. Phase Change Morphology and Thermal Cycling Stability

**Figure 7:**
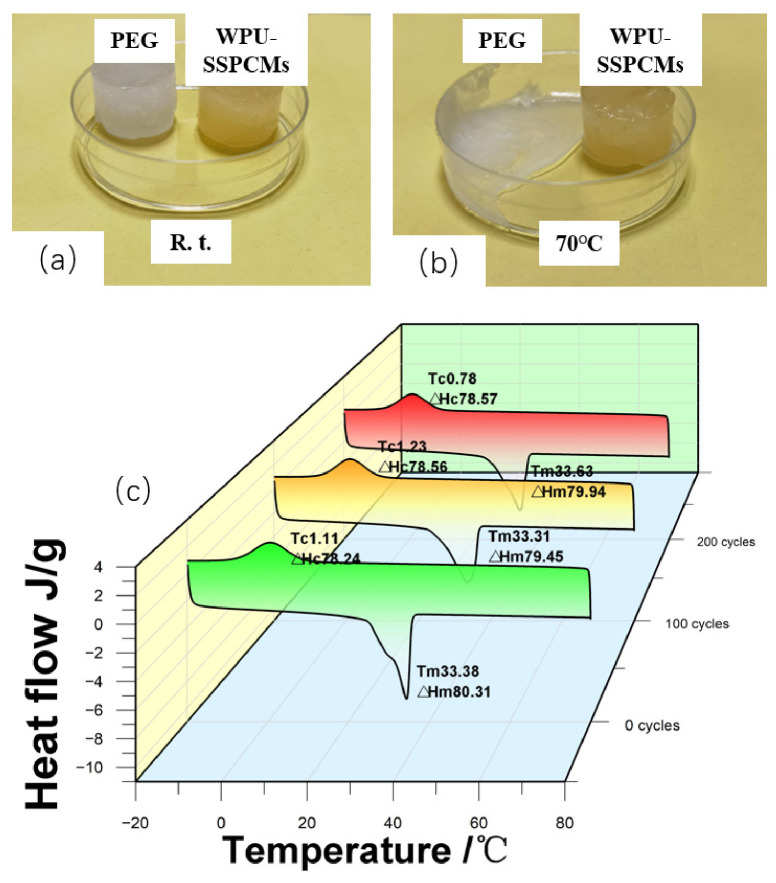
Morphological changes in PEG and WPU-SSPCMs at different temperatures: (**a**) room temperature and (**b**) 70 °C. (**c**) DSC thermograms and corresponding phase-change temperatures and enthalpies of WPU-SSPCMs before and after 100 and 200 thermal cycles.

To characterize the solid–solid phase transition behavior of WPU-SSPCMs, morphological changes in PEG and WPU-SSPCMs (molar ratio 1:2:0.67) were examined at varying temperatures; the test results are shown in Figure 7a,b. At ambient temperature, all specimens remained in the solid state. Upon heating to 70 °C for 20 min, PEG underwent complete melting, whereas WPU-SSPCMs preserved their solid morphology without any liquid exudation. This indicates that the crosslinking constraint exerted by the hard segments on the soft segments enables macroscopic solid–solid phase transition behavior. Additionally, to evaluate practical applicability, WPU-SSPCMs (molar ratio 1:2:0.67) were subjected to 100 and 200 heating–cooling cycles. DSC results (Figure 7c) demonstrate that the melting temperature and enthalpy values of the cycled samples exhibited minimal variation, with the heating and cooling curves nearly overlapping [35]. These results confirm that the WPU-SSPCMs maintain excellent thermal cycling stability even after 200 heating–cooling cycles, fulfilling the requirements for temperature-regulating textiles subjected to repeated temperature fluctuations.

### 3.7. Application on Cotton Fabric

**Figure 8:**
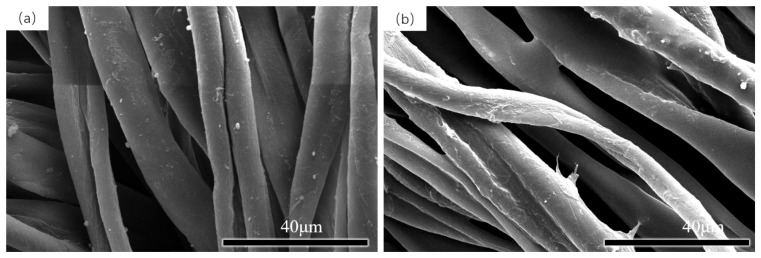
SEM images of the fabric surface. (**a**) Untreated fabric; (**b**) thermoregulating fabric.

The surface morphology of the thermoregulating fabric treated with 150 g·L^−1^ WPU-SSPCM was characterized by scanning electron microscopy (SEM), and the corresponding images are shown in Figure 8. As shown in Figure 8, the micrograph reveals that, relative to the untreated substrate, the fibers of the thermoregulating fabric are uniformly coated with a continuous film and exhibit localized protrusions and adhesive deposits. These findings substantiate the successful deposition of the WPU-SSPCM onto the fabric via the finishing treatment.

**Figure 9:**
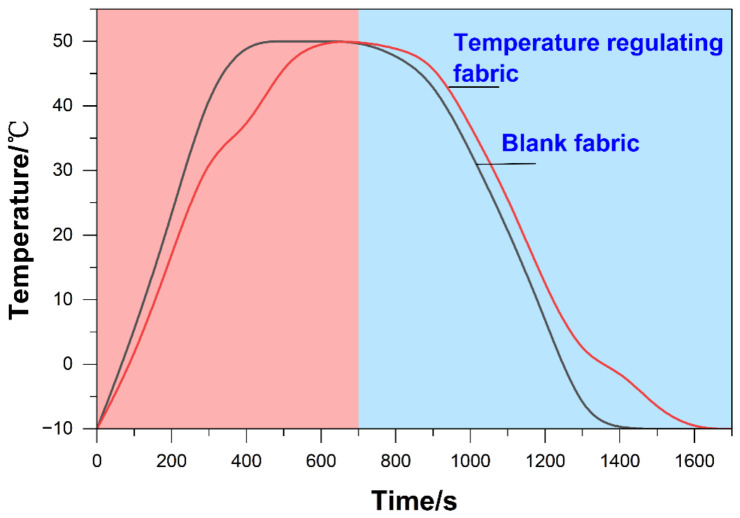
Comparison of the heating and cooling curves of fabrics before and after finishing treatment.

The fabricated WPU-SSPCMs were applied to cotton fabric to develop thermoregulating textiles, and the thermal regulation performance was assessed. The uncoated cotton fabric and the thermoregulating fabric were exposed to identical conditions and subjected to a heating–cooling cycle ranging from −10 °C to 50 °C and back to −10 °C. A digital thermometer was employed to continuously monitor and record the fabric temperature. The results (Figure 9) indicate that during the heating phase, the surface temperature of the uncoated fabric increased rapidly in response to the rising ambient temperature, whereas the thermoregulating fabric displayed a distinct thermal plateau near the melting point of the WPU-SSPCMs (approximately 33 °C), accompanied by a markedly reduced heating rate. This suggests that the WPU-SSPCMs absorbed thermal energy and underwent a phase transition, thereby retarding the temperature elevation on the fabric surface. Correspondingly, during the cooling phase, the thermoregulating fabric exhibited a thermal plateau near the crystallization temperature, indicating that the phase change material released stored latent heat and attenuated the temperature decline on the fabric surface [36]. These results demonstrate that the application of WPU-SSPCMs to cotton fabric effectively mitigates the influence of ambient temperature fluctuations on the fabric surface, thereby enhancing its thermal energy storage and thermoregulating performance.

To further investigate the durability performance of thermoregulating fabrics, the washing durability was evaluated by measuring the retention of phase change enthalpy after different numbers of washing cycles. The results are presented in Table 1.

Table 1 shows that the thermoregulating fabric exhibited an initial phase change enthalpy of 10.23 J·g^−1^ before washing. After 15 and 30 washing cycles, the enthalpy retention rates were as high as 96.85% and 92.91%, respectively. A gradual decrease in the retention rate was observed with increasing washing cycles. This decline can be attributed to the mechanical forces generated during washing, which caused partial dissolution and removal of the WPU-SSPCM coating from the fiber surface, thereby reducing the latent heat storage capacity of the fabric. Nevertheless, even after 30 washing cycles, the fabric retained 92.91% of its initial phase change enthalpy. These results demonstrate that the prepared thermoregulating fabric possesses excellent washing durability while maintaining outstanding thermal energy storage performance.

## 4. Conclusions

A PEG-based crosslinked network of WPU-SSPCMs were successfully synthesized, employing PEG as the soft segment, HMDI as the isocyanate, and GL as the chain extender. The results showed that the phase-change temperature and enthalpy of WPU-SSPCMs progressively increased with increasing PEG molecular weight, and PEG2000 was identified as the ideal soft segment for thermoregulating textile applications. Comparative investigations of the aliphatic isocyanates IPDI and HMDI revealed that HMDI imposed greater constraints on the soft segments, resulting in reduced phase-change temperatures and superior thermal stability, attributable to its highly symmetrical bicyclic structure and increased molecular rigidity. Furthermore, a chain-extension temperature of 70 °C facilitated a more effective and sustained monomeric interaction, thereby improving the thermal energy storage capacity of the materials. An increase in the monomer molar ratio corresponded to a proportional rise in hard segment content, thereby inducing reductions in both the melting temperature and phase change enthalpy. Under the optimized synthesis parameters, the obtained WPU-SSPCMs showed excellent latent heat storage capability, thermal stability, and cycling durability. Moreover, the treated cotton substrates demonstrated favorable thermal regulation performance, underscoring the potential of WPU-SSPCMs for integration into intelligent thermoregulating textiles. This study provides a feasible strategy for tailoring the molecular structure and thermal energy storage performance of PEG-based crosslinked WPU-SSPCMs. The findings offer valuable guidance for the design of solid–solid phase change materials for thermoregulated textile applications.

## Figures and Tables

**Table 1:** Effect of washing cycles on the thermal energy storage performance of fabrics.

	Influence of Enthalpy	△H_m/_J·g^−1^	Heat Retention Rate/%
Number of Washes			
0		10.23	100
15		9.91	96.85
30		9.50	92.91

## Data Availability

The original contributions presented in this study are included in the article. Further inquiries can be directed to the corresponding author(s).
